# Delayed Crosslinking Amphiphilic Polymer Gel System with Adjustable Gelation Time Based on Competitive Inclusion Method

**DOI:** 10.3390/polym11020381

**Published:** 2019-02-21

**Authors:** Bin Xu, Huiming Zhang, He Bian

**Affiliations:** 1 College of Chemistry and Chemical Engineering, Binzhou University, Binzhou 256600, China; zhanghuiming1982@163.com (H.Z.); hbiansn@163.com (H.B.); 2 Anhui Tianrun Chemical Industry Co., Ltd., Bengbu 233010, China

**Keywords:** delayed crosslinking, amphiphilic polymer gel, competitive inclusion, gelation time

## Abstract

Delayed crosslinking polymer gel systems are widely utilized in deep profile control processes for water production control in oilfields. In this paper, a kind of delayed crosslinking amphiphilic polymer gel system with adjustable gelation time based on competitive inclusion was prepared and its delayed crosslinking gelling properties were studied. The amphiphilic polymer of P(acrylamide (AM)–sodium acrylate (NaA)–*N*-dodecylacrylamide (DDAM)) was synthesized and it showed much better salt resistance, temperature resistance, and shear resistance performance compared with hydrolyzed polyacrylamide (HPAM). Phenol can be controlled released from the the cavity of β-cyclodextrin (β-CD) ring in the presence of the hydrophobic group used as the competitive inclusion agent in the amphiphilic polymer backbone. Accordingly, the gelation time of the delayed crosslinking amphiphilic polymer gel system is closely related to release rate of the crosslinker from the the cavity of β-CD ring. This study screened an amphiphilic polymer with good salt resistance and temperature resistance performance, which can be used in high temperature and high salinity reservoirs, and provided a feasible way to control the gelation time of the polymer gel system by the competitive inclusion method.

## 1. Introduction

Water production in oil-producing wells becomes more and more serious as the wells mature, and it is very important to control water production and improve oil recovery [1,2]. Among many profile control techniques, in-depth profile control methods have been developed quickly and practiced successfully such as cross-linked polymer microspheres [2,3] and delayed crosslinking polymer gel, which is widely used because of its high gel strength and good plugging effect [4,5,6]. 

There are two main issues that need further attention in the application of delayed crosslinking polymer gel systems: the gelation time and the gel strength. The gelation time determines the extent of gel penetration into formation layers when polymer gel system is injected, while the salt resistance and temperature resistance performance of the polymer determine the gel strength of the gels system. 

Adjustable gelation time is necessary in delayed crosslinking polymer gels systems during the profile control process. There are many ways to control the gelation time and the key is to control the release rate of the crosslinkers. The most commonly used polymer gel systems in profile control are hydrolyzed polyacrylamide (HPAM) and chromium (III) ions. Gels prepared with metallic crosslinkers such as chromium chromium, aluminum, and zirconium have lower stability at high temperatures and short gelation time, and are not profitable in high temperature reservoirs and deep profile control process [6,7,8,9]. The crosslinking ions can be formed into chelated ions to increase the gelation time, but there are limitations to the gelation strategy due to the lack of control over the release kinetics of chelated crosslinking ions [10]. Another popular water-based gel system for water-control applications is based on a phenol/formaldehyde crosslinker system [4,11,12]. The phenol/formaldehyde polymer gel system is thermally stable, and the gelation time is controllable over a wide temperature range [4]. Multiple emulsions can also be employed to increase the gelation time for the delayed crosslinking polymer gel systems [13,14].

The most commonly used polymer in the polymer gel systems for water shut-off is hydrolyzed polyacrylamide (HPAM). The viscosity enhancement of HPAM is mainly up to the expansion of solvated chains, owing to the repulsion of carboxylate groups. HPAM shows poor salt resistance, temperature resistance, and shear resistance performance, which hindered its application in high temperature and high salinity reservoirs [15,16,17,18]. As a result, many polymers are developed and applied in high temperature and high salinity reservoirs, such as modified xanthan [19], amphiphilic polymer like hydrophobically modified polyacrylamides (HMPAM), and so on. For example, the modified xanthan/chromium gels are stable up to at least 120 °C [19]. Compared with HPAM, HMPAM exhibits much better salt resistance, temperature resistance, and shear resistance performances; much higher thickening property; and good emulsification performance, and thus can enlarge both the swept volume and displacement efficiency [14,20,21,22,23,24,25,26,27,28,29].

Controlled release of organic compounds is most commonly reported in cyclodextrins (CDs) and CD polymers. Because of the hydrophobic and van der Waals interactions, the hydrophobic guests with a suitable molecular size for the CDs are capable of getting into the cavity of the CD ring to form CD inclusion complexes, which can realize various functional properties, including self-assembly, self-healing, drug release, removal of organics, and so on [30,31,32,33,34,35,36,37]. In the presence of competitive agents, the inclusion complexes can release the guest organics by competitive displacement of the inclusion molecule from its complex [37].

Currently, there are limitations to gelation strategies to form delayed crosslinking gels due to the lack of control over the release kinetics of crosslinkers. To address this challenge, we describe a new approach to form delayed crosslinking polymer gel using competitive displacement of the inclusion molecule from its CD complex. In this paper, a kind of delayed crosslinking HMPAM gel system was prepared based on competitive inclusion. The salt resistance, temperature resistance, and shear resistance performance of HMPAM compared with HPAM and the delayed crosslinking property of the HMPAM gel system were studied. The crosslinker was first involved in the cavity of β-cyclodextrin (β-CD) to form inclusion complex, and then the crosslinker in the inclusion complex was released as a result of the competitive inclusion of the hydrophobic group in the HMPAM solutions under certain pH circumstances to form the delayed crosslinking HMPAM gel with an adjustable gelation time.

## 2. Materials and Methods

### 2.1. Materials

Acrylamide (AM) was recrystallized twice from acetone and then dried under vacuum. Sodium acrylate (NaA) was formed by the reaction of acrylic acid (AA) with sodium hydroxide at the same molar ratio. The hydrophobic monomer *N*-dodecylacrylamide (DDAM) was synthesized by the reaction of acryl chloride and dodecyl amine. Sodium dodecyl sulfate (SDS) was recrystallized twice from ethanol. Potassium persulfate (KPS) and sodium bisulfite (SBS) were used as the initiators, which were recrystallized from deionized water. The crosslinkers used in this study were methenamine, phenol, and citric acid. β-CD is also an analytical reagent, and was recrystallized from water and then dried under vacuum. All aqueous solutions were prepared using deionized water. All reagents were all analytical reagents obtained from Sinopharm Chemical Reagent Co., Ltd. (Shanghai, China).

The hydrolyzed polyacrylamide (HPAM) was an industry product obtained from Anhui Tianrun Chemicals Co. Ltd. (Bengbu, China), with a molecular weight of about 1.5 × 10^7^ and a hydrolysis degree of about 25%.

### 2.2. Synthesis of Hydrophobically Modified Polyacrylamide

**Figure 1:**
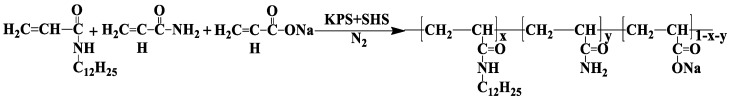
Synthesis of P(acrylamide (AM)–sodium acrylate (NaA)–*N*-dodecylacrylamide (DDAM)). KPS—potassium persulfate.

A kind of amphiphilic polymer of P(AM–NaA–DDAM) was synthesized using micellar copolymerization as reported [14]. The ratio for the three comonomers of AM, NaA, and DDAM was 74:25:1, so the hydrolysis degree of P(AM–NaA–DDAM) was 25%, the same as that of the HPAM employed in this study. The reaction temperature was 30 °C and the reaction time was 8 h, the overall concentration of monomers was 20 wt %, the concentration of the initiator KPS was 0.5 wt % relative to the total monomer feed while the molar ratio of SBS and KPS was 1:1, and the molar ratio of SDS to hydrophobic monomer (SMR) was 25. The synthesis of the HMPAM using AM, NaA, and the hydrophobic monomer DDAM as the comonomers is shown in Figure 1. The product was purified by excessive acetone to remove the residual monomers and SDS three times and then dried in vacuum at 45 °C for 48 h.

### 2.3. Rheology Measurement of Polymer Solutions and Polymer Gel Systems

The apparent viscosity of polymer solutions and polymer gel systems was measured using a NDJ-5S viscometer (Shanghai, China) at 6 rpm, 45 °C. Rheological measurements of polymer solutions were performed at 45 °C using an Anton Paar MCR301 stress-controlled rheometer (Graz, Styria, Austria) equipped with a concentric cylinder (outer diameter of 27 mm).

### 2.4. Intrinsic Viscosity Measurement and Molecular Weight Estimation for Polymer

The molecular weight of HPAM can be calculated by the Mark–Houwink equation according to China standard GB/T 12005.10-1992: determination for molecular weight of polyacrylamide by viscometry. In this study, $M=802{\left[\eta \right]}^{1.25}$, where [η] is the intrinsic viscosity of HPAM according to GBT12005.1-1989: determination for limiting viscosity number of polyacrylamide. A conventional Ubbelohde viscometer was used to obtain the intrinsic viscosity using the dilute method. Viscometry measurements were carried out in 1.00 mol g/L NaCl solutions at 30 °C. The initial polymer concentration was 200 mg/L. The polymer solutions were injected into the Ubbelohde viscometer and the flowing time through the quantitative ball was recorded three times for parallel until the flowing time difference was less than 0.2 s. The average value of the three times was chosen as the final data. HPAM and HMPAM are similar in structures, the mole fraction of hydrophobic comonomer in HMPAM was 1% and it does not significantly affect the intrinsic viscosity measurement, especially when the intromolecular interaction in HMPAM solutions was shielded. In this study, by using methyl-β-cyclodextrin to shield the hydrophobes from associating [38,39], HMPAM can be approximately viewed as HPAM and its molecular weigh can be also be calculated by the Mark–Houwink equation, as mentioned above.

### 2.5. Preparation of β-CD/Crosslinker Inclusion Complex

Both the crosslinkers of phenol and methenamine can be encapsulated into the CD cavity. In this study, β-CD/phenol complex was prepared. The inclusion complex of crosslinker/β-CD at the molar ratio of 1:1 was obtained by the method of saturated aqueous solution at 45 °C under gentle stirring. Formulation containing β-CD was first prepared at a certain concentration, and then the crosslinker was added to the formulation respectively until saturation and then stirred for 24 h to form inclusion complexes. The solid inclusion complexes were obtained after the solvent was evaporated to dryness at 45 °C.

### 2.6. Determination of Crosslinker Concentration during Controlled Released Process

Phenol concentration during the controlled released process in the presence of competitive inclusion agents was measured using 4-aminoantipyrine as a colouring agent combined with potassium ferricyanide in ammonium chloride buffer solution (pH = 10) and detected at 510 nm using a UV-visible spectrophotometer. The absorbance A and phenol concentration C (<20 mg/L) follows the following equation: A = 0.305C − 0.117, with a R^2^ value of 0.996.

### 2.7. Measurement of Gelation Time and Gel Strength

The HMPAM solutions were prepared by dissolving P(AM–NaA–DDAM) in deionized water with gentle stirring at room temperature for 12 h and then the solutions were kept for24 h at 45 °C to remove air bubbles. The delayed crosslinking HMPAM gel systems were prepared by mixing HMPAM solutions, the inclusion complex and other crosslinkers of a certain concentration at 45 °C. The apparent viscosity of the gel systems was measured using a NDJ-5S viscometer (Shanghai, China) at 6 rpm as the gel strength. Gelation time is defined as the time required for the gelling components to form a gel. The gelation time was determined by viscometer method in this study and was identified by the time at which the viscosity of the gel solution suddenly increases in the correlative curve between viscosity and time [40]. 

## 3. Results and Discussions

### 3.1. Solution Properties of P(AM–NaA–DDAM)

**Figure 2:**
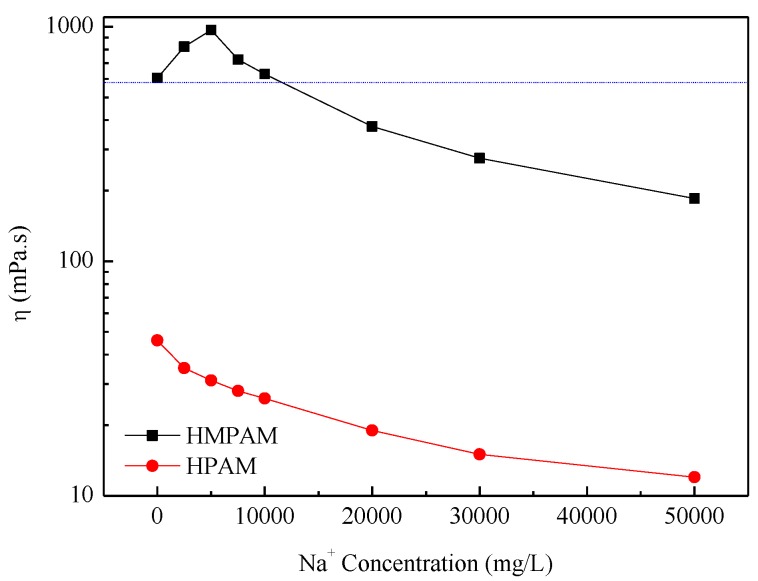
The apparent viscosity of polymer solutions with 1500 mg/L versus Na^+^ concentration at 45 °C. HMPAM—hydrophobically modified polyacrylamides; HPAM—hydrolyzed polyacrylamide.

**Figure 3:**
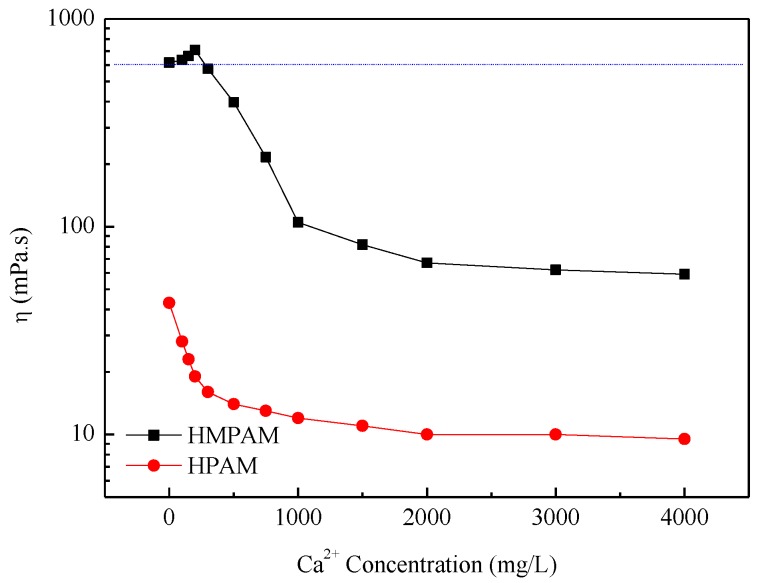
The apparent viscosity of polymer solutions with 1500 mg/L versus Ca^2+^ concentration at 45 °C.

The critical aggregation concentration (CAC) of P(AM–NaA–DDAM) is about 800 mg/L as, already reported in our previous study [14]. Above CAC, the HMPAM showed good thickening properties. The large increase in viscosity was caused by extensive intermolecular association, which can lead to network structures in HMPAM solutions [20,21,22,23,24,25,26,27,28,29]. The apparent viscosity of HMPAM solutions and HPAM solutions is shown in Figure 2 and Figure 3. Obviously, HMPAM of P(AM–NaA–DDAM) showed much better salt resistance performance both in sodium chloride and calcium chloride solutions compared with HPAM. Besides, P(AM–NaA–DDAM) exhibited salt-thickening behavior at certain sodium and calcium ion concentrations. In the absence of salt, the charge moieties of NaA incorporated along the polymer backbone have two opposing effects on the viscosity for HMPAM. The intramolecular electrostatic repulsion leads to chain repulsion and viscosity enhancement, while the intermolecular charge repulsion hinders the hydrophobic association of hydrophobic groups and yield to viscosity reduction. In the presence of salt, stronger intermolecular hydrophobic interactions promote the physical crosslinking network and lead to viscosity enhancement of HMPAM. However, at high salt concentrations, the strong associating microstructures cause a collapse of the crosslinking network structure and viscosity reduction [21,22,23].

The maximum viscosity was shown at a lower concentration using CaCl_2_ compared with NaCl, which is probably because divalent cations such as Ca^2+^ have a higher ratio of cation charges to cation surface area and at high Ca^2+^ concentration, the shielding effect on the electrostatic resistance among ions leads to polymer coil overlap [21,22]. As to HPAM, the increasing salinity leads to viscosity reduction due to the double layer compression effect by cations.

**Figure 4:**
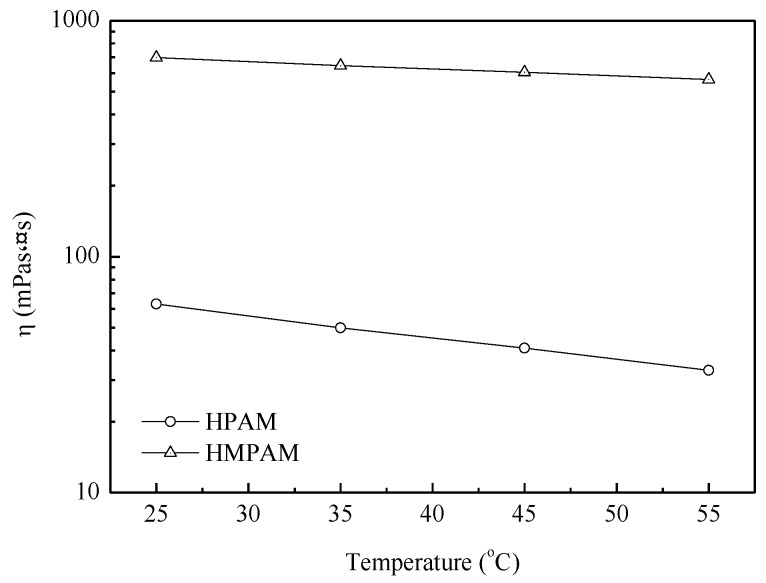
The apparent viscosity of polymer solutions with 1500 mg/L versus temperature at 45 °C.

As shown in Figure 4, HMPAM demonstrated better temperature resistance performance compared with HPAM. Temperature has two effects on the HMPAM viscosity; namely exothermic hydrophobic hydration and endothermic hydrophobic-hydrophobic interaction. As a result of these two effects, the viscosity of HMPAM decreased smoothly from 25 to 55 °C, while HPAM viscosity decreased sharply with the increasing temperature [21,24].

**Figure 5:**
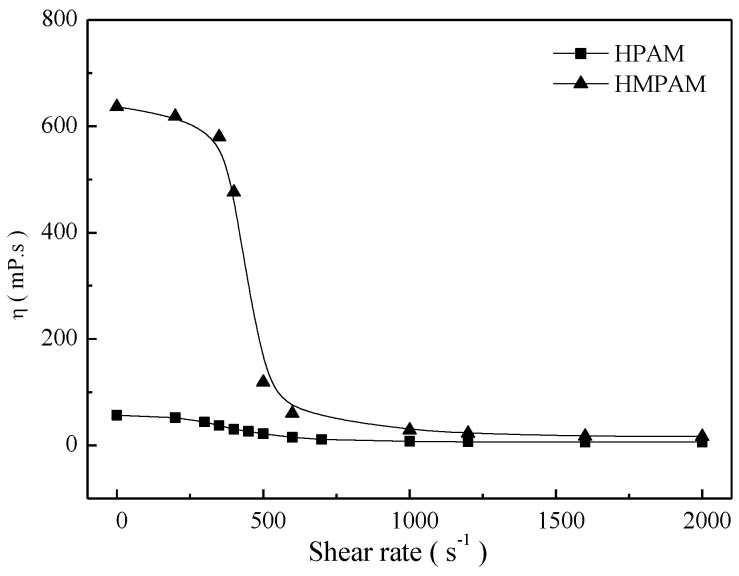
The shear behavior of polymer solutions with 1500 mg/L at 45 °C.

As shown in Figure 5, both polymer solutions exhibited a Newtonian plateau at low shear rates and shear thinning behavior at higher shear rates. For HMPAM solutions, the viscosity finally tended to be constant at high shear rates as a result of the balance between intermolecular association and disassociation. These results indicate that HMPAM has a perfect shear tolerance at high shear rates.

**Figure 6:**
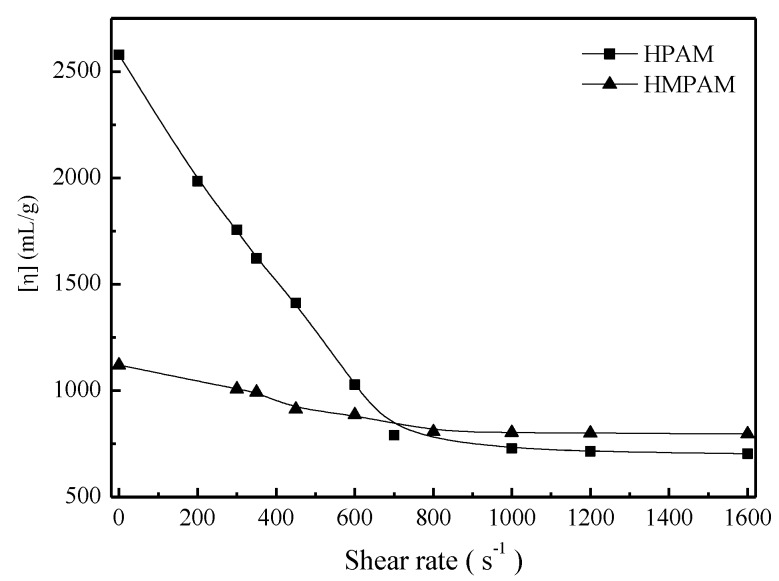
Intrinsic viscosity of polymers versus shear rate at 45 °C.

**Figure 7:**
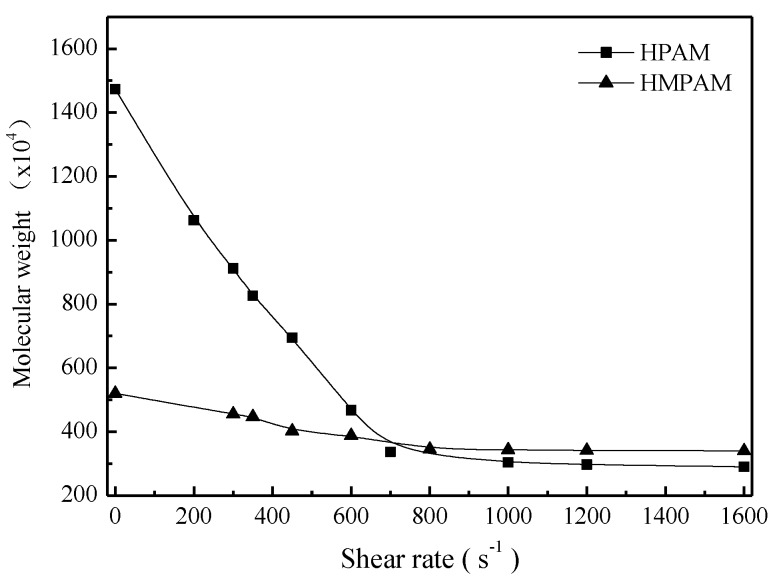
Molecular weight of polymers versus shear rate at 45 °C.

To better explain shear tolerance performance of HPAM and HMPAM, both polymer solutions with 1500 mg/L were sheared at a certain shear rate for 10 min and then the intrinsic viscosity measurement and molecular weight calculation were conducted as described in Section 2.4. Figure 6 and Figure 7 describe the intrinsic viscosity and the molecular weight of both HMPAM and HPAM as a function of shear rate. The intrinsic viscosity and molecular weight of HPAM decreased dramatically with the increasing shear rates, which indicated that irreversible mechanical degradation on molecular weight occurred for the HPAM molecule. As to HMPAM, mechanical degradation did not obviously occur during the shear process because the dissociation process of the physical crosslinking network structure is reversible and the hydrophobic groups can form new association after removing the shear [27,28,29].

### 3.2. Gelling Properties of the Delayed Crosslinking HMPAM Gel System

The formulation of the delayed crosslinking amphiphilic polymer gel system utilized in this study was similar to that reported in our previous study [14]: 0.15% P(AM–NaA–DDAM), 0.3% methenamine, 0.02% phenol (or 0.26% phenol/β-CD inclusion complex), and 0.3% citric acid.

**Figure 8:**
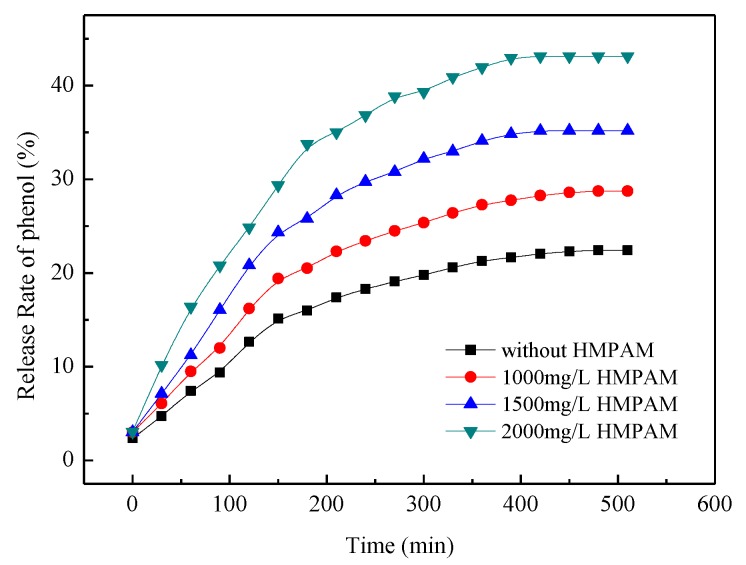
Release rate of phenol from β-cyclodextrin (β-CD)/phenol inclusion complex at 45 °C.

Phenol can be released from the cavity of the CD ring when the solid phenol/β-CD inclusion complex is dissolved in water, especially in the presence of competitive agents with stronger hydrophobicity. The release rate of phenol from the inclusion complex is shown in Figure 8. The controlled release of phenol is a dynamic equilibrium process. The release rate of phenol depends on the stability constant of cydodextrin inclusion complexes and the molar ratio of competitive inclusion agent to phenol. The release rate of phenol was about 23% without competitive inclusion agents after 8 h. The release rate of phenol increased with the increasing HMPAM concentration. The results showed that phenol can be released in a controlled manner from the cavity of the β-CD ring in the presence of hydrophobic group of dodecyl group by changing the mole ratio of dodecyl group and phenol.

**Figure 9:**
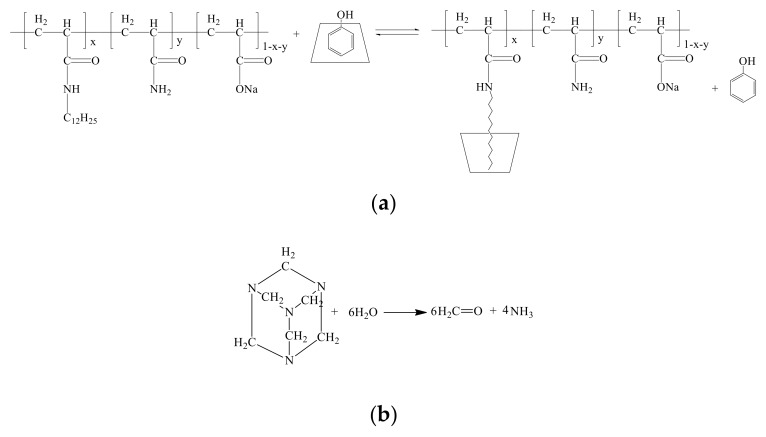

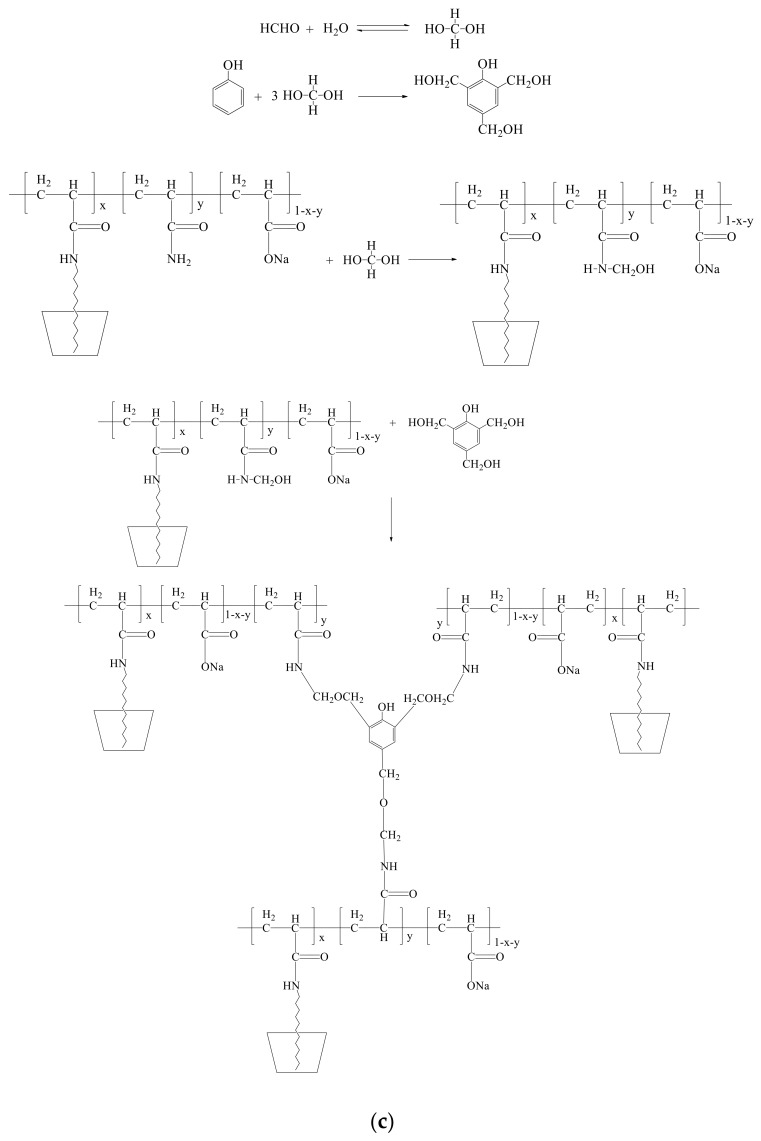
The probable gelation mechanism of the delayed crosslinking amphiphilic polymer gel system based on competitive inclusion method. (**a**) Controlled release of phenol in the presence of amphiphilic polymer; (**b**) formaldehyde release from methenamine; (**c**) gelation mechanism of the HMPAM/phenolic resin gel system.

As to the delayed crosslinking amphiphilic polymer gel system based on competitive inclusion method, the probable gelation mechanism can be depicted in Figure 9. Phenol can be controlled released from the cavity of CD in the presence of amphiphilic polymer and formaldehyde can be released from methenamine as shown in Figure 9a,b, respectively; then, acrylamide groups in HMPAM and phenol-formaldehyde can react to form gel [41], as shown in Figure 9c. 

**Figure 10:**
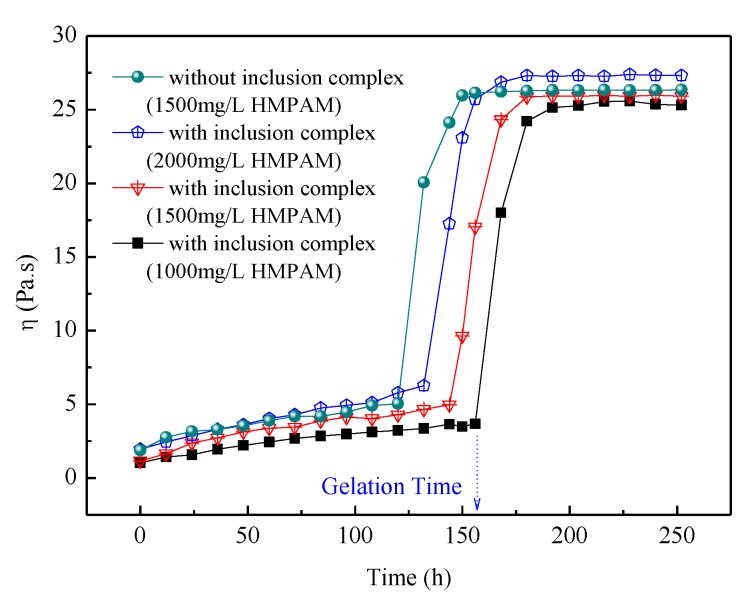
Influence of competitive agent on apparent viscosity of the gel system.

The influence of the competitive agent on apparent viscosity of the delayed crosslinking HPAM gel is shown in Figure 10. The gelation time of the delayed crosslinking HMPAM gel system is closely related to the release rate of phenol. Without the competitive inclusion method, the gelation time of HMPAM gel system was about 120 h, whereas using the hydrophobic group as the competitive inclusion agent, the gelation time was about 144 h with 1500 mg/L HMPAM. The gelation time of delayed crosslinking HMPAM gel system decreased with the increasing HMPAM concentration. It can be also seen that the final gel strength depends mainly on HMPAM concentration for the delayed crosslinking HMPAM gel system. The adjustable delayed gelation time could help the gel system to achieve in-depth profile control for water production control. However, further study on the release kinetics of crosslinkers from CD cavity in the presence of competitive inclusion agents and how to precisely control the gelation time of the delayed crosslinking polymer gel system is still needed.

## 4. Conclusions

A kind of delayed crosslinking amphiphilic polymer gel system with adjustable gelation time based on competitive inclusion method was prepared and its delayed crosslinking gelling properties were studied. The amphiphilic polymer of P(AM–NaA–DDAM) showed much better salt resistance, temperature resistance, and shear resistance performance compared with HPAM. The gelation time of the delayed crosslinking amphiphilic polymer gel system is closely related to the release rate of the crosslinker from the the cavity of the β-CD ring. This study provided a feasible way to control the gelation time of the polymer gel system by the competitive inclusion method.
